# An Embroidered Electrochemical Sensor to Measure Glucose Made with Commercially Available Textile Materials

**DOI:** 10.3390/bios15020109

**Published:** 2025-02-14

**Authors:** Marc Martínez-Estrada, Ignacio Gil, Raúl Fernández-García

**Affiliations:** Departament of Electronic Engineering, Escuela Superior de Ingenierías Industrial, Aeroespacial y Audiovisual, Universitat Politecnica de Catalunya, c/Colom 1, 08222 Terrassa, Spain; ignasi.gil@upc.edu (I.G.); raul.fernandez-garcia@upc.edu (R.F.-G.)

**Keywords:** electrochemical, sensor, flexible, textile, glucose

## Abstract

A textile electrochemical sensor manufactured with commercially available textile materials is presented to determine glucose concentration. The sensor design consists of three electrodes manufactured with two different conductive yarns, one made with a silver coating and the other with stainless steel fibres. Different combinations of them are used to prepare three different electrochemical textile sensor combinations. The first sensor is built only with silver-coated yarn and used as a reference sensor. The other two sensors are prepared with different combinations of conductive yarns. The textile sensors perform a cyclic voltammetric test, where it is demonstrated that the glucose concentration over the sensor can be related with the increase in the current measured. The results allow us to identify feeding voltages where the concentration–current relation is close to linear. The textile sensor shows a sensitivity between 0.0145 and 0.0452 μA/(mg/dL) for the 45–180 mg/dL glucose concentration range and 0.0012 and 0.0035 μA/(mg/dL) for the 180–1800 mg/dL range for the different sensor types presented. The regression coefficients for the sensitivities range between 0.9266 and 0.9954. This research demonstrates the feasibility to develop a fully integrated textile electrochemical sensor made completely with commercially available textile materials.

## 1. Introduction

In the recent decade, wearable systems have been promoted by researchers due to the wide range of possibilities they offer to end users. Electrochemical wearable systems based on flexible substrates, such as textile sensors, have been reported as a promising option for real-time healthcare, sports, and environmental applications [1,2,3,4,5,6,7]. Researchers have been exploring new types of sensors that can be implemented in textiles, including electrochemical sensors.

Glucose and lactate sensors are two of the most widely used electrochemical sensors in healthcare and sports around the world. The importance to control the concentration of glucose and lactate in blood is high. It enables us to prevent hypoglycaemia or hyperglycaemia and determine the level of effort during training.

Both of them could be measured through sweat, which can provide an equivalent concentration in blood. The correlation between indicators such as glucose or lactate in blood and sweat concentration has been proven in several works [3,8,9]. The researchers in [9] have proved, with a large group of subjects, that blood glucose values are equal to the sweat glucose level after 8 min. They showed that by using the 8 min shift on the linear regression, a correlation coefficient value equal to 0.92 is obtained.

Obtaining electrochemical textile glucose sensors has been one of the focuses of researchers, due to the potential improvements in quality of life that it could provide to people with diabetes around the world. Nowadays, people with diabetes use invasive methods to obtain the levels of glucose in blood, such as a finger-prick test or electronic blood sugar monitor, comprising taking data from interstitial blood through a needle introduced into the skin [10,11]. In addition, the integration of electrochemical glucose sensors on textile substrates has other positive characteristics, such as being introduced into clothing, mass manufacturing, good relationships with patients, stretchability, and adhesion to the skin without disturbances.

Electrochemical textile sensors used to measure glucose, lactate or chemical components on fluids have been investigated in recent years [12,13,14,15,16,17]. The electrode material has high importance with regard to the behaviour of electrochemical sensors. In this sense, most sensors have been developed using specific materials during manufacture, such as gold yarns coated with Ag/AgCl, enzymatic treatments (lactate/glucose oxidase), or polyester yarns coated with Ag/AgCl and carbon inks [18,19,20].

In terms of textile electrochemical sensor research, two different kinds of sensors are observed: enzymatic [7,18,21,22,23,24] and non-enzymatic [25]. Electrochemical glucose sensors based on enzymatic treatments use the glucose oxidase (GOx) response to measure the glucose concentration in a sample. Enzymes react to the glucose oxidation reduction by increasing the electric response on the working electrode, which is commonly the place where enzymes are located. The case of the embroidered electrochemical sensors presented in the work of [23] shows how textile technology is prepared to integrate conductive manufactured yarns to build a sensor over a shirt. However, to achieve the presented functionality, they need to prepare conductive yarns with a silver or carbon ink coating and, additionally, treat the carbon working electrode with glucose oxidase for glucose detection. The applicability of yarns manufactured with an enzymatic treatment should be demonstrated during production in the textile industry, along with their ability to maintain their properties through the manufacturing processes.

Focusing the attention on the non-enzymatic textile electrochemical sensor [25], some researchers have manufactured a cuprous oxide/conductive fabric prepared to be used as a working electrode on an electrochemical sensor. The results demonstrated the functionality, stability and accuracy of the working fabric electrode to be used as part of an electrochemical sensor. In this work, the focus of our research is to build an electrochemical sensor based on a textile substrate with commercially available materials without any additional enzymatic or chemical treatment. As it is observed during the literature review, enzymatic textile electrochemical sensors have been developed more during recent years than non-enzymatic electrochemical textile sensors. The designed sensor was manufactured by means of technologies and materials available from the textile industry, without any additional treatments or processes that are not available in large-scale industrial production. The presented work aims to demonstrate the actual possibilities of the textile and electronics industries for building a functional electrochemical textile sensor.

This paper is organised as follows: Section 2 describes the materials and methods used, including the conductive yarn properties, the textile substrates and their implementation, as well as the measurement set-up and methodology. In Section 3, the experimental results are shown, discussed and analysed. Finally, in Section 4, the conclusions of this study are presented.

## 2. Materials and Methods

**Figure 1:**
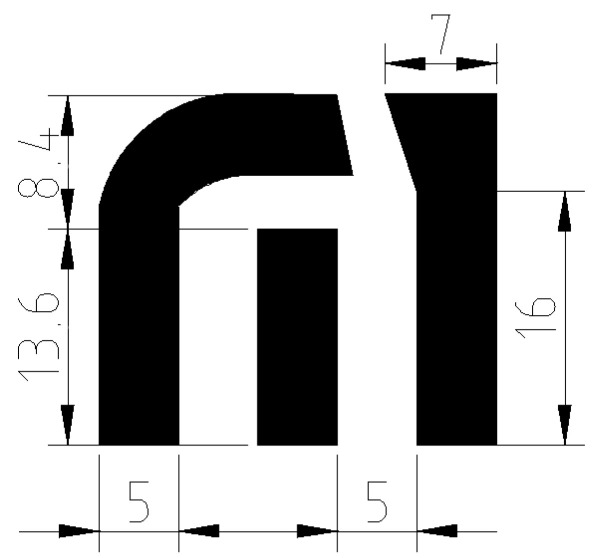
Proposed glucose-embroidered sensor with dimensions in mm.

The proposed glucose sensor is based on a three-electrode embroidered structure. The three electrodes are defined as being a counter electrode (CE), working electrode (WE) and reference electrode (RE) (Figure 1). The CE and WE electrodes are in charge of the voltage feeding of the sensor. Meanwhile, through RE and WE electrodes, the current and voltage are measured. The WE is a common electrode, as it takes part in the measurement and the feeding procedure. The material used for the CE and WE should be sensitive to chemical substances and the material for the RE should provide electrical stability. The variety of materials used in the literature is extensive for WEs and CEs; however, in the case of the RE, the most commonly used material is Ag/AgCl.

In this paper, the impact of electrode-conductive materials on sensor behaviour was evaluated using two different conductive yarns. Firstly, a commercial 117/17 dtex 2-ply was chosen [26]. This yarn was made of polyamide (PA) multifilament, which are coated with pure silver. Secondly, a commercial Bekaert yarn was chosen. The Bekaert yarn is made of stainless steel fibres mixed with polyester fibres in a composition of 40% and 60%, respectively [27]. These yarns were tested using durability tests in a previous study, proving their functionality and resistance during daily use [28,29]. The electrical properties for both yarns are presented in Table 1 as follows:

The glucose sensor was embroidered over a felt substrate, which was selected due to its dimensional stability and absorbance. The substrate has a thickness of 0.886 mm and a dry permittivity of 1.32.

**Figure 2:**
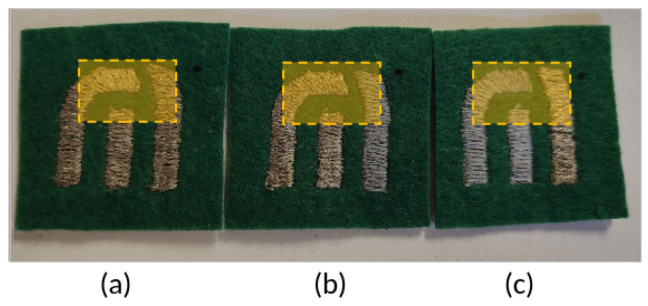
Electrochemical sensor built with different materials. The shaded square marks the detection zone. (**a**) Sensor Type A, (**b**) Sensor Type B, and (**c**) Sensor Type C.

To manufacture the sensors, some considerations are made. WEs and CEs were embroidered with the same yarn since they were used as feeding electrodes. Meanwhile, the RE was embroidered with the other kind of yarn. Based on these requirements and materials used, three types of sensors were defined, and they are summarized in Table 2. Sensor Type A was fabricated entirely with silver-coated yarns on all of its electrodes, as a reference sensor; Sensor Type B used silver-coated yarns for the CE and WE, and stainless steel yarns for the RE. Sensor Type C was built with stainless steel yarns for the CE and WE, and silver-coated yarns for the RE. Figure 2 shows the different manufactured electrochemical sensors.

The next step of the test plan was to prepare glucose solutions. Five different solutions were prepared based on the most common concentrations found in the literature [21,22]. The glucose $\left(C_{6}H_{12}O_{6}\right)$ used was supplied by Montpket & Esteban SA (Barcelona, Spain), with a molar mass of 180.156 kg/mol. The solutions are listed in Table 3. On the one hand, concentrations between 45 and 180 mg/dL are interesting because they indicate the usual blood sugar concentration range for a person with diabetes, covering a range of values indicative of hypoglycaemia to hyperglycaemia [30]. On the other hand, concentrations from 180 mg/dL up to 1800 mg/dL are used as a control of the sensor functionalities and capabilities.

The process followed starts with cleaning the containers with distilled water and isopropyl alcohol, with the objective of removing any impurities that are present. The glucose quantities for each solution are weighed and dropped into the containers. Additionally, 100 mL of distilled water is used as a solvent and poured into the containers. At this moment, each solution is mixed for a few seconds until the glucose is completely dissolved, and the mixing procedure is repeated for each solution for each the tested sensor.

Five samples of each sensor type are prepared to measure the five different solutions. As a result, a total of fifteen sensors were prepared.

**Figure 3:**
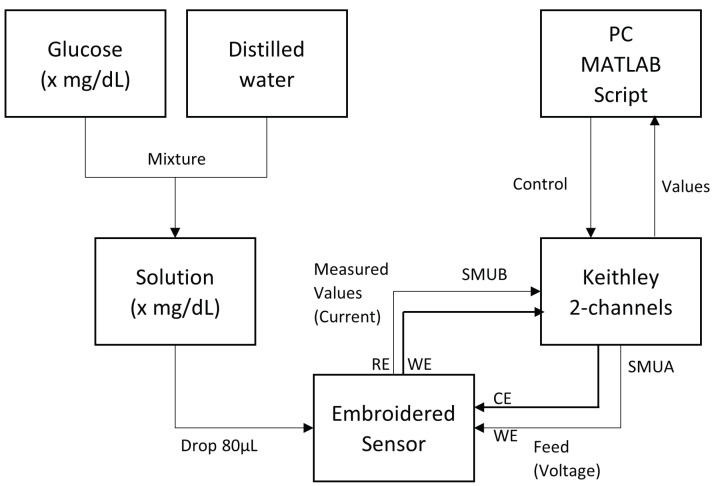
Set-up scheme of the measurement process. The scheme shows the solution drop preparation (left column in scheme) and the set-up of the measurement and data part (right column in scheme).

**Figure 4:**
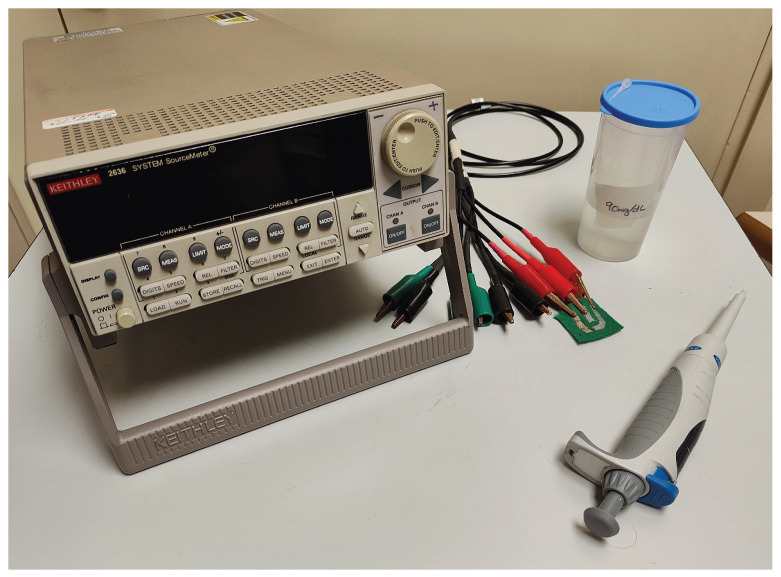
Experimental set-up. The SMU system is connected to the sensor, prepared for the solution drop over the sensing zone.

A Keithley 2636B Source Meter (SMU) from Keithley Instruments (Solon, OH, USA; EE.UU) was used to measure the electrical response of the sensor. To perform the different tests, two SMU channels of the Keithley were set up as feeding and measure channels, respectively. The proposed sensor was connected as follows: the WE and CE were connected to the feed channel (SMU A) and the WE and RE were connected to the measurement channel (SMU B). Figure 3 shows the set-up for a cyclic voltammetry measurement, and Figure 4 shows the experimental set-up for a single measurement.

The test performed is conducted as follows: The tested sensor is placed on a clean surface, where its electrodes are connected to the SMUs (Figure 3). The Source Meter is connected with a laptop, with a MATLAB (v. r2024b) script being utilized to set-up the initial state of the test and perform the cyclic voltammetry measurement.

**Figure 5:**
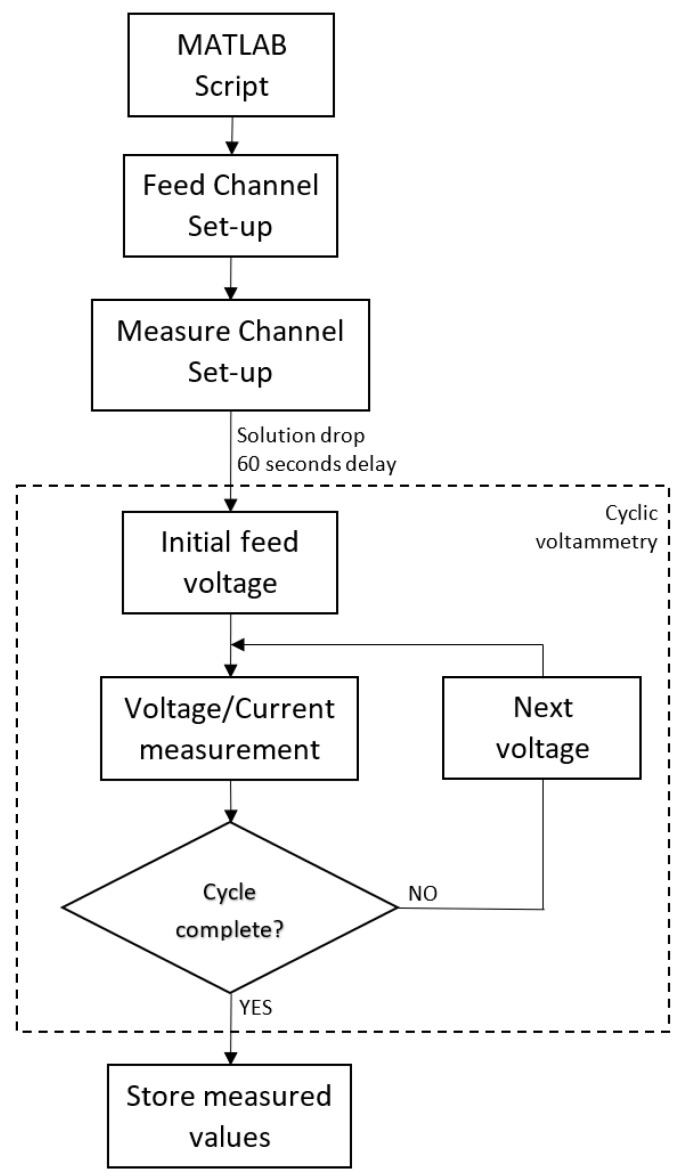
Flux diagram of the MATLAB script for a visual depiction of the steps performed to obtain cyclic voltammogram data.

The solution is prepared to be dropped over the sensor; in this case, 80 μL of solution is being poured over the sensor. The procedure continues with the execution of the MATLAB script, which performs a voltage sweep from −200 mV to 200 mV with a 50 mV/s scan rate, and a set-up measurement range between 1 nA and 10 μA. The voltage range is selected as being the coincidence voltage range, where lactate and glucose effects on sensors can be observed simultaneously. The procedure is schematically depicted in Figure 5.

Note that the script includes a 60 s delay, which is the time selected to wait between the solution drop over the sensor and the start of the cyclic test measurement, ensuring that the solution is absorbed completely by the substrate.

## 3. Results and Discussion

**Figure 6:**
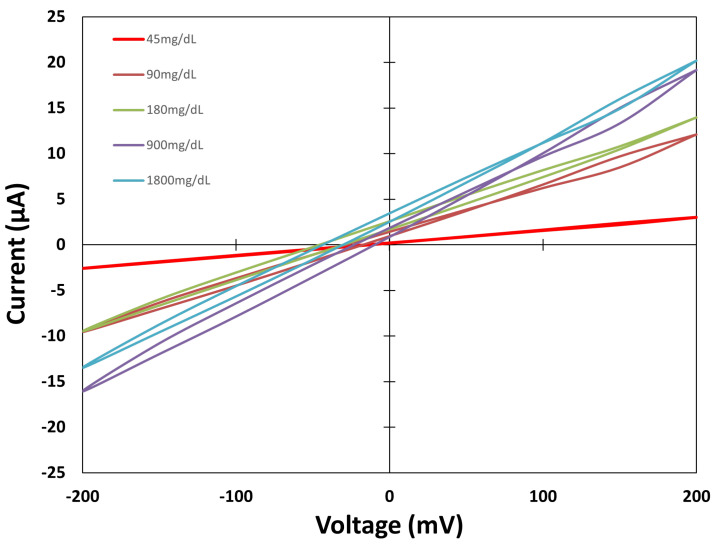
Cyclic voltammetric values for Sensor Type A.

**Figure 7:**
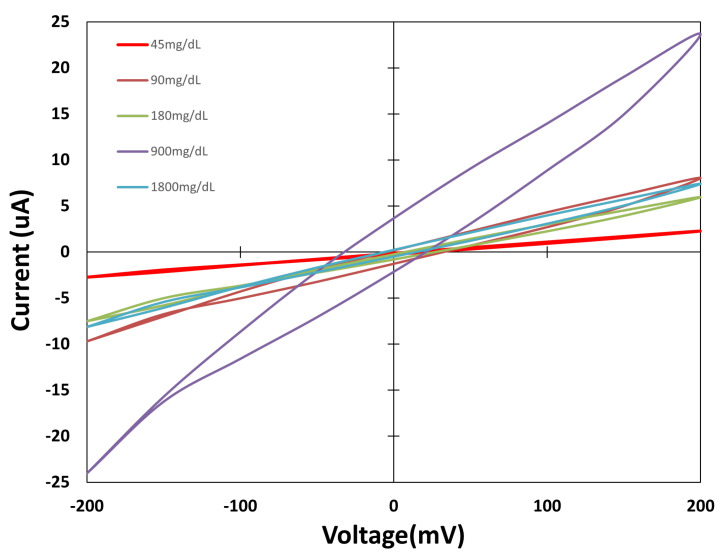
Cyclic voltammetric values for Sensor Type B.

**Figure 8:**
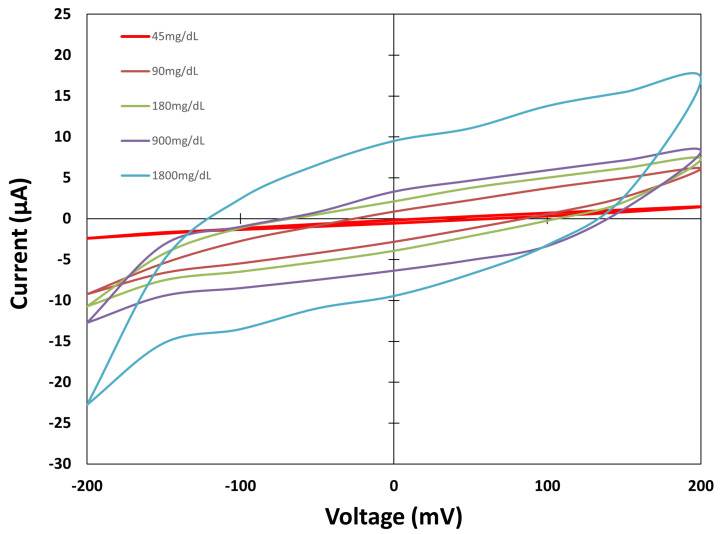
Cyclic voltammetric values for Sensor Type C.

The sensors are placed one by one on the test surface and connected to both SMUs. The system is switched on, and the MATLAB script is prepared. The drops are poured onto the sensor zone, and the measurement is performed through the MATLAB script, which waits 60 s at the beginning, as stated earlier. The three different types of sensors are measured. Figure 6, Figure 7 and Figure 8 show the values obtained for each group against different concentration solutions when a cyclic voltammetric is performed.

### 3.1. Cyclic Voltammetric Study

Figure 6 shows the embroidered electrochemical sensor values from the sensor made only with Shieldex material (Sensor Type A). The results show narrow cycles, which indicates that oxidation and reduction currents are similar, due to the three electrodes being manufactured with the same material. This can also be explained by the low Faradaic current effect provoked by the oxidation reduction processes that Ag/AgCl electrodes can detect. During the voltage sweep, the current absolute value increases as the concentration in solutions increases. This behaviour is mainly observed from −50 to 200 mV.

Figure 7 shows the Sensor Type B group of sensors where the CE and WE are build with silver yarn (Shieldex), with stainless steel yarn being used for the RE. The cyclic voltammetric test shows that the cycles are not correlated with the concentrations, due to the higher resistance of the stainless steel material used in the RE. Moreover, as stainless steel is not as stable as silver against chemical reactions, it does not provide the stability needed on the reference electrode, which could result in unreliable tension being applied on the reaction. On the working electrode, which is made of silver in this case, the aim is for the reaction to occur over it. However, if the silver material presents stable behaviour, no reaction occurs on working electrode surface. The observed results exclude Sensor Type B as a glucose detector. Therefore, Sensor Type B behaviour cannot be analysed further.

Figure 8 shows the values measured from the Sensor Type C group of sensors. This design shows a more recognisable cyclic voltammetric shape. The Faradaic currents are higher on the electrodes of Sensor Type C, and the values increase as the solutions’ concentration increases., denoting the difference in the oxidation and reduction processes for different concentrations. As a result, the current measured increases as the concentration increases. After the presentation of the cyclic voltammetric curves for the three cases studied, the effect of capacitive currents is observed on the lowest concentration. The values measured for the 45 mg/dL concentration are slightly different between the three sensor types, showing the capacitive background currents present on the sensors. As the solution concentration increases, the Faradaic currents provoked by the oxidation reduction processes increase; however, the effect of capacitive currents is still present on the sensors.

### 3.2. Lineal Regression Study

The dependence of the current measured with a glucose concentration is deeply analysed for Sensor Types A and C in this section. The cyclic voltammetric results provide the information to locate the best feeding voltage. Specifically, the voltage ranges for each electrochemical sensor presented higher linear behaviour. Sensor Type A was analysed at 50 mV and 100 mV voltage points, while Sensor Type C behaviour was analysed at 50 mV and −50 mV. Both linear regression studies also present the confidence zone for each regression group for a 95% confidence interval.

**Figure 9:**
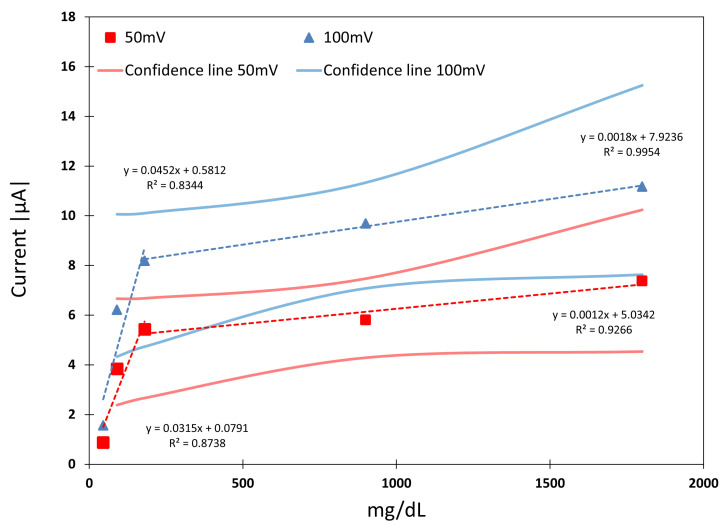
Linear regression of Sensor Type A for the feeding voltages selected. Confidence lines are represented as continuous lines.

Figure 9 shows the sensor current against glucose concentration. It is observed that the sensor’s behaviour can be linearised in two sections: lower concentration values, up to 180 mg/dL, and higher concentration values, between 180 mg/dL and 1800 mg/dL. At a higher glucose concentration, linear dependence is observed along with a regression coefficient ($R^{2}$) of up to 0.9954 for 100 mV feeding. Meanwhile, at a lower concentration, the linearity it is reduced, achieving $R^{2}$ between 0.8344 and 0.8738 at 100 mV and 50 mV feeding voltages, respectively.

**Figure 10:**
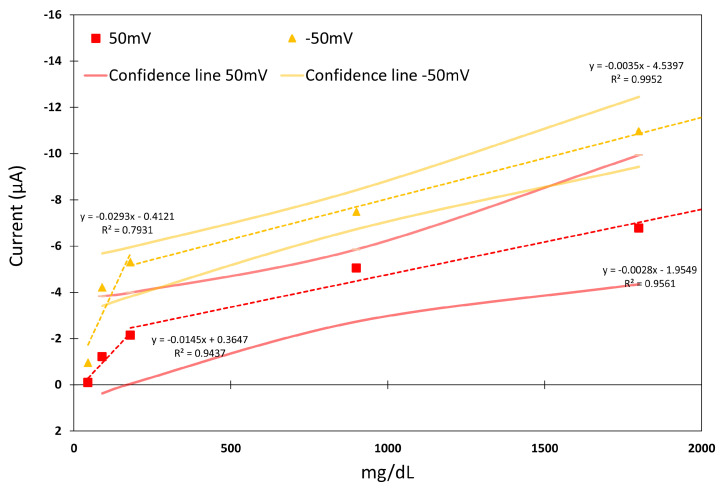
Linear regression of Sensor Type C for the feeding voltages selected. Confidence lines are represented as continuous lines.

Figure 10 shows the Sensor Type C linear regression study for voltages of 50 mV and −50 mV, which were observed to be the more sensitive feeding voltages according to the results in Figure 7. Regarding Sensor Type A, two different tendencies, above and below 180 mg/dL, can be observed for each feeding voltage. The results denote a better linear dependence for higher concentration values with a $R^{2}$ of up to 0.9952 for −50 mV feed voltage.

Table 4 summarises the linear regression study for a 50 mV feed voltage. Despite the sensor’s linearity depending on the feeding voltage for each case, the results denote a clear dependence of the measured current with the glucose concentration. It is demonstrated that the functionality of the proposed electrochemical sensor developed only with commercial textile materials without any additional enzymatic or chemical treatments.

After the linear regression study, some of the literature sensor data were compared with the proposed work. Table 5 shows the most important research studies found. In them, electrochemical textile or flexible sensors with higher manufacturing similarity to the presented sensor can be observed. Only other electrochemical textile sensors can be found without any chemical treatments, with the manufacturing material being used as a working electrode on an electrochemical sensor with two common industrial electrodes. The presented work shows the possibility to manufacture a complete electrochemical sensor over a textile or flexible substrate with available textile industrial materials, achieving values of range detection and sensitivity similar to other electrochemical sensors found in the literature.

## 4. Conclusions

In this paper, we presented a three-electrode electrochemical embroidered sensor to measure various chemical concentrations, such as glucose, using only commercially available conductive yarns. Also, we did not use any chemical or enzymatic treatments to increase sensitivity. Three different designs were tested when changing materials of the different electrodes. The sensor with stainless steel yarn on RE electrodes was excluded as an electrochemical sensor as its cyclic voltammetric measurement did not show any correlation between the concentration of the solution and current measured due material selection. The other two sensor topologies, which used silver yarn on RE electrodes, can be used to correctly determine glucose concentrations. However, the sensor implemented with stainless steel on the WE and CE electrodes showed a wider curve in cyclic voltammetric, which indicates a higher sensitivity to the oxidation reduction process (Faradaic current). Sensor Type C showed good linear regression, with sensitivities between 0.0028 and 0.0145 μA/(mg/dL) for the different concentration ranges with a regression value ($R^{2}$) between 0.9437 and 0.9561. The results denote the feasibility to develop a fully integrated textile electrochemical glucose sensor made completely using commercially available textile materials, avoiding any enzymatic or chemical treatments.

## Figures and Tables

**Table 1:** Electrical properties of the yarns used.

	Shieldex Yarn	Bekaert Yarn
Linear resistance (Ω ${\mathrm{cm}}^{-1}$)	<30	50
Conductivity $\left(10^{6}{\mathrm{Sm}}^{-1}\right)$	1.28–1.32	62

**Table 2:** Materials used to manufacture the different sensor types.

Sensor Type	CE	WE	RE
A	Silver	Silver	Silver
B	Silver	Silver	Stainless Steel
C	Stainless Steel	Stainless Steel	Silver

**Table 3:** Solutions prepared with glucose for the sensor test.

mM	g Glucose	Concentration mg/dL
0.25	0.045	45
0.5	0.09	90
1	0.18	180
5	0.9	900
10	1.8	1800

**Table 4:** Linear regression obtained for each of the designed sensor tested at a 50 mV feeding voltage.

Group	Concentration mg/dL	Sensitivity μA/(mg/dL)	$R^{2}$
Sensor Type A	45–180 180–1800	0.0315 0.0021	0.8738 0.9266
Sensor Type C	45–180 180–1800	0.0145 0.0028	0.9437 0.9561

**Table 5:** Comparison table with the most similar electrochemical flexible/textile sensors in the literature.

Material	Flexible Electrodes	Treatments	Concentration mM	Sensitivity (μA/mM)	Ref
$Cu_{2}O$-coated	WE	-	0–0.4	3937	[25]
Pt-MWCNTs/CSF	WE	GOx	0–5	288.86	[22]
Graphene	WE	GOx	0.1–0.5	85	[31]
PDMS/CNT/PPY	WE	GOx	0–12	10	[32]
Ag/AgCl Carbon paste	RE/WE/CE	GOx	0–1	2.276	[7]
Ag/AgCL-Au	RE/WE/CE	GOx	0–0.5	11.7	[24]
Ag/AgCl Carbon ink	RE/WE/CE	GOx	0–40	-	[23]
Au	RE/WE/CE	GOx	0.01–100	0.126	[18]
Ag-SS	RE/WE/CE	-	0–10	0.63	This work

## Data Availability

Data are contained within the article.
